# Emerging role of FBXO22 in carcinogenesis

**DOI:** 10.1038/s41420-020-00303-0

**Published:** 2020-07-27

**Authors:** Jiangting Cheng, Min Lin, Man Chu, Longyuan Gong, Yanli Bi, Yongchao Zhao

**Affiliations:** 1 Key Laboratory of Combined Multi-Organ Transplantation, Ministry of Public Health, First Affiliated Hospital, Zhejiang University School of Medicine, Hangzhou, China; 2 Institute of Translational Medicine, Zhejiang University School of Medicine, Hangzhou, China; 3 The Second Affiliated Hospital and Yuying Children’s Hospital of Wenzhou Medical University, Wenzhou, Zhejiang, China

**Keywords:** Ubiquitylation, Ubiquitin ligases

## Abstract

The F-box protein 22 (FBXO22), one of F-box proteins, has been identified to be critically involved in carcinogenesis. FBXO22 promotes proliferation in breast cancer and lung cancer, but suppresses migration and metastasis. FBXO22 exerts oncogenetic functions via promoting the ubiquitination and degradation of its substrates, including KDM4A, KDM4B, methylated p53, p21, KLF4, LKB1, Snail, CD147, Bach1, PTEN, and HDM2. FBXO22 is also regulated by several regulatory factors such as p53, miR-155, SNHG14, and circ_0006282. In this review, we summarize the regulatory factors and downstream targets of FBXO22 in cancers, discuss its functions in tumorigenesis, and further highlight the alteration of FBXO22 expression in a variety of human malignancies. Finally, we provide novel insights for future perspectives on targeting FBXO22 as a promising strategy for cancer therapy.

## Facts

FBXO22 targets multiple substrates for ubiquitination and degradation.

FBXO22 is critically involved in tumorigenesis and tumor progression.

FBXO22 might be a therapeutic target for cancer treatment.

## Open questions

Which targets of FBXO22 are pivotal for cancer development and malignant progression?

Do E3 liagses regulate the protein levels of FBXO22?

How the inhibitors of FBXO22 could be developed and discovered for cancer therapy?

## Introduction

Post-translational modification (PTM) is one of the critical pathways in regulation of cellular events such as proliferation, apoptotic death, cell cycle, mitosis, motility, and innate immunity^1–3^. PTMs include, but are not limited to, ubiquitination, phosphorylation, acetylation, methylation, succinylation, and sumoylation^4–6^. Among these PTMs, ubiquitination is one of the most studied and is mediated by ubiquitin proteasome system (UPS) to trigger protein degradation. In general, three enzymes are involved in UPS-induced protein degradation, including ubiquitin activating E1 enzyme, ubiquitin conjugating E2 enzyme, and ubiquitin E3 ligase^7^. The target protein is labeled by ubiquitins and subsequently degraded by the 26S proteasome complex, leading to reduction of substrate protein. E3 ligases recognize and recruit the target protein for ubiquitination, thus they were extensively characterized^8^. Among E3 ligases, Cullin-RING E3 ligase (CRL) complex is one of the largest families, including CRL1–3, 4A, 4B, 5, 7, and 9^9–11^. CRL1, also known as SKP1-cullin 1-F-box protein (SCF) E3 ligase complex, contains cullin-1 acting as the scaffold protein, RBX1 for recruiting ubiquitin-loaded E2, SKP1 working as an adaptor protein to connect F-box protein, and F-box protein for selecting substrates for degradation^12^. A total of 69 F-box proteins encoding by human genome, are divided into three subclasses according to their variable domains: FBXW proteins with WD40 repeat domains, FBXL proteins with leucine-rich repeat domains, and FBXO proteins with other domains like kelch repeats or proline-rich domains^13,14^.

F-box proteins have been validated to play a pivotal role in carcinogenesis and progression^13,15^. They are involved in various physiological and pathological processes which include proliferation, apoptosis, metastasis, cancer stem cell generation, epithelial–mesenchymal transition (EMT), drug resistance in human cancers^16–18^. It is important to notice that F-box proteins, including FBXO22, have oncogenic or tumor suppressive role in cancer development and progression. In the following sections, we will describe the regulatory factors and downstream targets of FBXO22 in a variety of human cancers, and the alteration of its expression levels in human cancer tissues.

## Upstream regulators of FBXO22

The p53 protein as a traditional tumor suppressor has been identified to be mutated in a variety of human malignancies^19^. Mutant p53 proteins lose anticancer function due to impaired cellular homeostasis and damaged genome stability, leading to enhancement of survival, invasion, and metastasis^19,20^. Evidence has demonstrated that p53-mediated tumor suppressive activity is in part through regulation of downstream targets and multiple signaling pathways^21^. One study validated that wild-type p53 increases the transcription of FBXO22 via binding to DNA and promotion of histone acetylation at FBXO22 promoter^22^. Specifically, p53 overexpression elevated FBXO22 mRNA level by real-time RT-PCR analysis. Data from chromatin immunoprecipitation (ChIP) on chip analysis demonstrated that FBXO22 is a direct p53 target^22^. Since FBXO22 might target numerous substrates for degradation or inactivation, p53 exerts its tumor suppressive activity partly via induction of FBXO22 expression. Another study reported that miR-155 could target FBXO22 in anterior uveitis^23^. It has been well known that miRNAs are small, non-coding RNAs that regulate hundreds of target genes at the post-transcriptional level, so that miRNAs govern multiple biological functions, such as differentiation, proliferation, stemness and oncogenesis^24^. Through TargetScan online computational algorithm and validation by a luciferase reporter gene assay, FBXO22 is identified as a target of miR-155^23^. Several studies have revealed that miR-155 plays an essentical role in oncogenesis and progression. For example, miR-155 promoted cell growth and invasion via regulation of epidermal growth factor receptor (EGFR) and nuclear factor-kappa B (NF-κB) in salivary adenoid cystic carcinoma^25^. One group reported that miR-155 was highly expressed in sera of hepatocellular carcinoma (HCC) patients, which is associated with blood telomerase level^26^. In addition, miR-155 inhibited proliferation, migration, invasion and triggered cell cycle arrest and apoptosis via regulating the expression of collagen triple repeat containing 1 (CTHRC1) in human melanoma^27^. Moreover, exosome-mediated miR-155 delivery led to cisplatin resistance of oral squamous cell carcinoma (OSCC) cells via induction of EMT^28^. However, it is unclear whether miR-155 targets FBXO22 in human cancer cells, which is required to further determine.

**Fig. 1:**
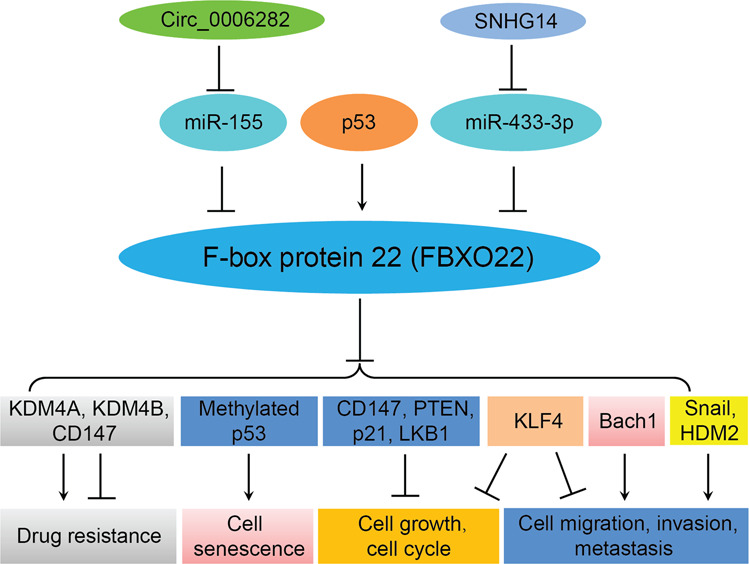
The regulation of FBXO22 and its downstream substrates. FBXO22 targets several substrates for ubiquitination and degradation, and is regulated by multiple regulatory factors. FBXO22 regulates proliferation, cell cycle, senescence, migration, invasion, metastasis, and drug resistance in human cancers.

Small nucleolus RNA host gene 14 (SNHG14), one long noncoding RNA, was reported to act as a competing endogenous RNA (ceRNA) to decoy miR-433-3p and subsequently increase FBXO22 expression in osteosarcoma cells^29^. Downregulation of FBXO22 or SNHG14 inhibited proliferation, motility of osteosarcoma cells, but stimulated apoptosis^29^. Token together, SNHG14 enhanced osteosarcoma progression through modulation of miR-433-3p/FBXO22 pathway. Recently, circular RNA (circRNA) circ_0006282 was revealed to facilitate tumor progression via sponging miR-155 to increase FBXO22 expression in gastric cancer^30^. Specifically, circ_0006282 functions as a ceRNA to spong miR-155 and cause the upregulation of its target, FBXO22, resulting in enhancement of proliferation and metastasis of gastric cancer cells^30^ (Fig. 1).

## Downstream targets of FBXO22

Accumulated evidence has demonstrated that FBXO22 targets several substrates for uiquitination and degradation (Table 1 and Fig. 1). The histone lysine demethylase 4 (KDM4) subfamily includes four proteins, KDM4A, KDM4B, KDM4C and KDM4D, which control chromatin structure and gene expression^31^. KDM4A, also termed as JMJD2A and JHDM3A, can demethylate histone H3 lysine 9 (H3K9) and 36 (H3K36) and H1.4K26, leading to regulation of genome replication and stability^31^. KDM4A is identified as a substrate of FBXO22^32^. Therefore, FBXO22 is potentially involved in development, differentiation and cancer via controlling KDM4A stability, leading to regulation of H3K9 and H3K36 methylation, which are important factors to maintain normal cellular function^32^. Similalry, KDM4B degradation is mediated by FBXO22 in breast cancer cells, resulting in regulation of selective estrogen receptor modulators (SERMs) activity, leading to modulation of tamoxifen resistance in ER-positive breast cancer cells^33^. FBXO22 is required for cell growth inhibition induced by tamoxifen, and FBXO22-induced KDM4B degradation is necessary for the antagonistic function of SERMs in breast cancer^33^. One study showed that FBXO22-KDM4A act as an E3 ubqiquitin ligase to govern methylated p53 stability via its degradation, leading to regulation of senescence^34^. In line with this, *Fbxo22*^*−/−*^ mice exhibited an increase of p53 expression level, and mouse embryonic fibroblast (MEFs) from *Fbxo22*^*−/−*^ mice had increased methylated p53, suggesting that FBXO22 might regulate the amount of methylated p53^34^.

**Table 1:** FBXO22 targets substrates for degradation in human diseases.

Substrates	Cell lines	Functions	Refs
KDM4A	HeLa, 293T, 293T-Rex	Regulation of cell cycle, involves in development, differentiation, cancer	^32^
KDM4B	MCF7, T47D	Tomaxifen resistance	^33^
Methylated p53	HeLa, U2OS, MCF7, 293T, HCA2, MEFs, HCT116, RPE-1	Regulating senescence	^34^
p21	HL-7702, HepG2, Huh7, Hep3B, Bel-7402, HLF, LM3, 293T	Promotes proliferation and tumor growth	^35^
KLF4	HepG2, Huh7, Hep3B	Promotes proliferation and invasion	^38^
LKB1	H322, H446, H460, H661, H1299, BT549	Promotes cell growth	^43^
CD147	293T, A549, SMMC-7721, Huh-7	Inhibits cisplatin resistance	^45^
Bach1	A549, H2009, 293T, KP, KPK.	Inhibits migration and metastasis	^47^
PTEN	293T, HeLa, SW620, SW480, LS174T	Promotes tumor growth	^48^
Snail	MDA-MB-231, Hs578T, MCF-7, ZR-75-1, T47D	Inhibits migration, invasion, and metastasis; promotes proliferation	^39^
HDM2	HeLa, MDA-MB-231, BT-549, 4T1	Inhibits invasion and metastasis	^42^

FBXO22 elevated proliferation of HCC cells and enhanced tumor growth in mice. Knockdown of FBXO22 in HLF and HepG2 cells led to suppression of proliferation and inhibition of colony formation, whilest overexpression of FBXO22 in LM3 and Hep3B cells promoted cell viability and colony formation^35^. Moreover, results from both subcutaneous and orthotopic mouse models showed that downregulation of FBXO22 slowed down the tumor growth in vivo^35^. Mechansitically, FBXO22 interacted with p21 and subsequently ubiquitinated p21 via its F-box domain for degradation. Strikingly, FBXO22 accelerated cell growth partly and modulated cell cycle progression via downregulation of p21. Consistently, FBXO22 expression was negatively associated with p21 level in HCC tumor samples^35^.

Kruppel-like factor 4 (KLF4) has tumour suppressive functions in a variety of human malignancies^36^. KLF4 is often downregulated in tumor specimens and associated with poor survival in cancer patients^37^. It has been shown that FBXO22 interacts with and destabilizes KLF4 via ubiquitination in HCC cells, leading to promotion of proliferation and invasion^38^. A negative correlation between FBXO22 and KLF4 was observed in HCC tumor samples^38^. Interestingly, FBXO22 was reported to exhibit a different function in breast tumorigenesis and metastasis^39^. Overexpression of FBXO22 elevated proliferation and facilitated colony formation in vitro and in vivo, but inhibited EMT, cell motility and invasion as well as metastasis in breast cancer^39^. Moreover, FBXO22 targets Snail, a key factor to trigger EMT process, for ubiquitination and degradation in a glycogen synthase kinase 3β (GSK-3β) phosphorylation-dependent manner. It is worth noting that Snail/Slug and ZEB-1/SIP1 families not only control EMT process, but also inhibit cell cycle progression by repression of cyclin D^40,41^. This study indicated that FBXO22 may act as an upstream regulator and play a dual role in mammary cancer by inducing Snail degradation: promotion of proliferation and suppression of metastasis^39^. Human homolog of mouse double minute 2 (HDM2) is often highly expressed in various types of cancers. The stability of HDM2 oncoprotein is regulated by FBXO22 by ubiquitin-dependent degradation in breast cancer cells^42^. FBXO22 targets HDM2 for ubquitination and degradation, leading to inhibitory effects on invasion and metastasis in breast cancer^42^. Consistently, FBXO22 level is negatively associated with HDM2 expression in patients with breast cancer^42^.

Liver kinase B1 (LKB1), a serine/threonine kinase, has been identified to involve in oncogenesis in various types of human cancers. LKB1 expression level is regulated by FBXO22 via proteasome-mediated degradation in non-small cell lung cancer (NSCLC) cells^43^. FBXO22 interacts with and triggers LKB1 for K63-mediated ubiquitination, leading to inhibition of LKB1 activity and subsequent inactivation of AMP-activated protein kinase (AMPK) and mammalian target of rapamycin (mTOR) signaling pathways^43^. FBXO22-mediated inactivation of LKB1 causes promotion of cell growth via modulation of AMPK and mTOR pathways in NSCLC cells^43^. CD147 as a transmembrane glycoprotein is often overexpressed in human malignancies and is involved in chemoresistance in cancer cells^44^. FBXO22 could ubiquitinate CD147 and result in its degradation, leading to enhancement of cisplatin sensitivity in lung cancer cells^45^. The transcription factor BTB and CNC homology 1 (Bach1) plays a regulatory role in cell cycle, senescence, angiogenesis, immunity, and carcinogenesis and metastasis. Bach1 expression is linked to recurrence of breast cancer patients, and Bach1 promotes migration and invasion in colon and prostate cancer cells^46^. Recently, FBXO22 was identified to mediate the Bach1 degradation and inhibit migration in lung cancer cells^47^. More recently, in agreement with the oncogenic role of FBXO22, phosphatase and tensin homolog on chromosome 10 (PTEN), a bona fide tumor suppressor, is validated as a direct substrate of FBXO22^48^. FBXO22 ubiquitinates and degrades nuclear PTEN via proteasome-mediated degradation in colorectal cancer, leading to tumor development^48^ (Table 1 and Fig. 1).

Multiple studies have dissected that FBXO22 could regulate the expression of several downstream targets, such as hypoxia-inducible factor (HIF1α), vascular endothelial growth factor A (VEGFA), tissue inhibitor of matrix metalloproteinase-1 (TIMP-1) and metalloproteinase-9 (MMP-9) in human cancer cells^49,50^. FBXO22 downregulation in melanoma cells suppressed migration, invasion and angiogenesis, and decreased the formation of blood vessels in nude mice^49^. Moreover, FBXO22 promoted the motility of melanoma cells and angiogenesis through upregulation of HIF1α and VEGFA^49^. In RCC cells, FBXO22 has no any effect on proliferation, but FBXO22 restricted RCC cell motility and reversed EMT via an increase of the activity of TIMP-1 and an decrease of MMP-9 expression as well as a reduction of VEGF secretion^50^. In line with this in vitro result, in vivo study showed that FBXO22 inhibited RCC metastasis. Altogether, FBXO22 mainly reduced migration, invasion and metastasis in RCC through suppression of MMP-9 and VEGF pathways^50^. These paradoxical findings suggest that the role of FBXO22 in metastasis is in a context dependent manner. Thus, future investigations should be directed to elucidate the molecular mechanisms how FBXO22 regulates VEGF pathway.

## Functions of FBXO22 in tumorigenesis

FBXO22 performs its functions via targeting its substrates by proteasome-mediated degradation in human malignancies, and exhibits its functions in controlling proliferation, cell cycle, apoptosis, migration, invasion, and metastasis. Numerous studies have been conducted to determine the role of FBXO22 in carcinogenesis by in vitro and in vivo experiments. The expression level of FBXO22 in various types of cancers has also been determined. To clarify the physiological function of FBXO22, the *Fbxo22* knockout mice have been established using the CRISPR-Cas9 approach. Two *Fbxo22*^−/−^ mice were viable, but had smaller size with the body weight reduced by 50% at six months of age, as compared to *Fbxo22*^+/+^ or *Fbxo22*^+/−^ mice^34^. Although most *Fbxo22*^−/−^ mice died within two days after birth, the genotype distribution of the offsprings from intercrossing *Fbxo22*^*+/−*^ mice is consistent with the Mendelian-based ratio of 1:2:1, indicating that *Fbxo22* is dispensable for early embryonic development^34^. Given that most *Fbxo22*^*−/−*^ mice died within a couple of days after birth, it is of high demanding in the field to generate conditional knockout mouse model (*Fbxo22*^*fl/fl*^) to investigate the role of Fbxo22 in tumorigenesis. Specifically, *Fbxo22* is deleted in various organs in combination with other established genetically modified mouse tumor models such as tumor suppressor inactivation (*Pten*^*fl/fl*^, *p53*^*fl/fl*^, or *Lkb1*^*fl/fl*^), particularly PTEN, p53, and LKB1 acting as the substrates of FBXO22, or oncogene activation (e.g. *KRas*^*G12D*^). *Fbxo22* deletion promoting or blocking tumorigenesis in these mouse models will elucidate the physiological role of FBXO22 in tumorigenesis in a given organ. In the following paragraphs, we will summarize the alteration of FBXO22 levels and its association with carcinogenesis and progression.

## FBXO22 expression in human tumor tissues

One study measured the expression of FBXO22 on a tissue microarray with 110 pairs of HCC specimens by immunohistochemistry (IHC) approach and indicated that FBXO22 is highly expressed in tumors, compared to adjacent non-tumor tissues^35^. Another study also observed the overexpression of FBXO22 in HCC tumor tissures^38^. Notably, FBXO22 expression level is correlated with serum AFP, tumor size, and vascular invasion. Furthermore, high expression of FBXO22 is associated with poor prognosis in patients with HCC^35^. High FBXO22 expression is also observed in melanoma tissues, compared with normal skin tissues^49^. Additionally, FBXO22 mRNA level is increased in lung squamous cell carcinoma and lung adenocarcinoma according to the data from the cancer genome atlas (TCGA) database^43^. Moreover, IHC staining result indicated that higher expresson of FBXO22 existed in lung adenocarcinoma tissues than adjacent normal tissues. The result of Western blotting analysis confirmed the increased FBXO22 expression in lung cancer tissues as well^43^. In support of the oncogenic role of FBXO22 in lung cancer, the high expression of FBO22 is correlated with poor overall survival in lung cancer patients^43^.

On the other hand, FBXO22 also exhibits tumor-suppressing characteristics. Lower expression of FBXO22 is associated with worse prognosis in estrogen receptor (ER)-positive and human epidermal growth factor receptor type 2 (HER2)-negative breast cancer patients^33^. Although one group found that FBXO22 expression is increased in primary breast tumor specimens^39^, FBXO22 expression is correlated with favorable clinical outcomes in patients with breast cancer^39^. Similarly, low expression of FBXO22 is associated with poor survival in patients with breast cancer^42^. Intriguingly, FBXO22 is downregulated in pregnancy-associated breast cancer via analysis of NCBI-GEO datasets^51^. IHC analysis in renal cell carcinoma (RCC) tissues found that FBXO22 expression levels were decreased in RCC specimens compared with those in normal renal tissues^50^. Lower expression of FBXO22 in RCC patients is associated with tumor size, TNM stage, and poor survival^50^.

**Fig. 2:**
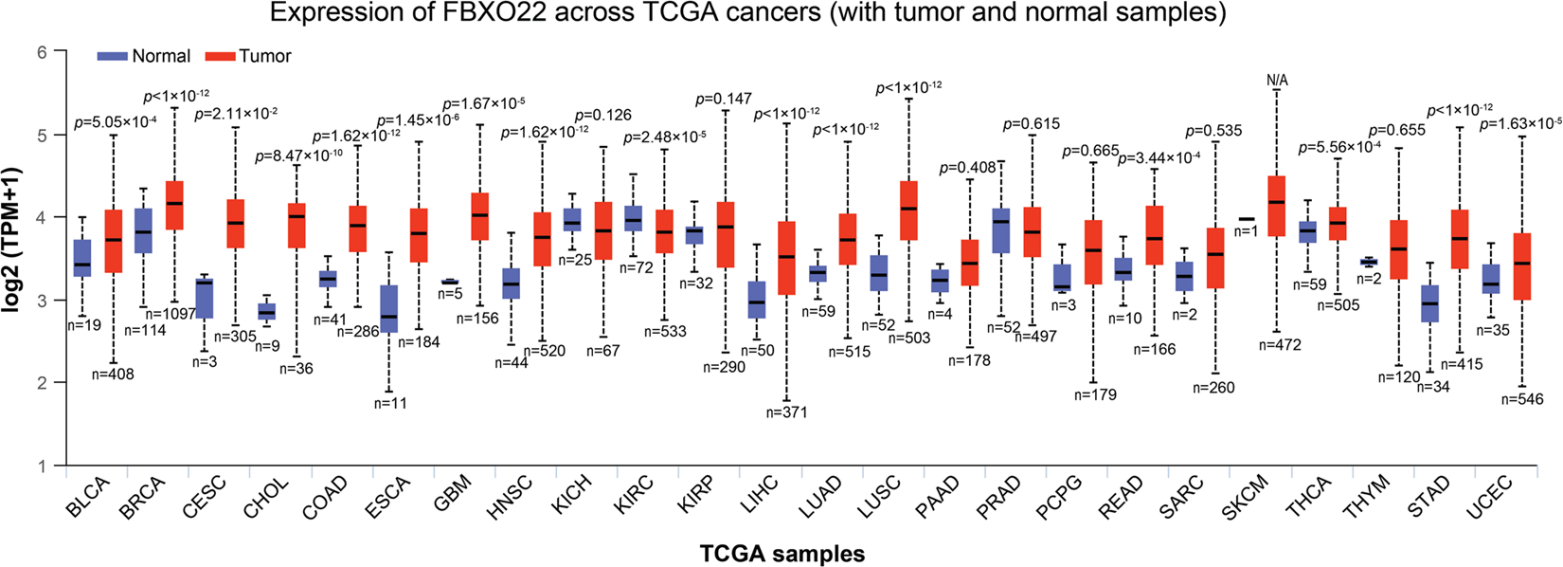
The expression of *FBXO22* in human tumor and normal tissues. The height of bar represents the mRNA expression of FBXO22 in TCGA cancer types. TPM transcripts per million. BLCA bladder urothelial carcinoma, BRCA breast invasive carcinoma, CESC cervical squamous cell carcinoma and endocervical adenocarcinoma, CHOL cholangio carcinoma, COAD colon adenocarcinoma, ESCA esophageal carcinoma, GBM glioblastoma multiforme, HNSC head and neck squamous cell carcinoma, KICH kidney Chromophobe, KIRC kidney renal clear cell carcinoma, KIRP kidney renal papillary cell carcinoma, LIHC liver hepatocellular carcinoma, LUAD lung adenocarcinoma, LUSC lung squamous cell carcinoma, PAAD pancreatic adenocarcinoma, PRAD prostate adenocarcinoma, PCPG pheochromocytoma and paraganglioma, READ rectum adenocarcinoma, SARC sarcoma, SKCM skin cutaneous melanoma, THCA thyroid carcinoma, THYM thymoma, STAD stomach adenocarcinoma, UCEC uterine corpus endometrial carcinoma.

**Fig. 3:**
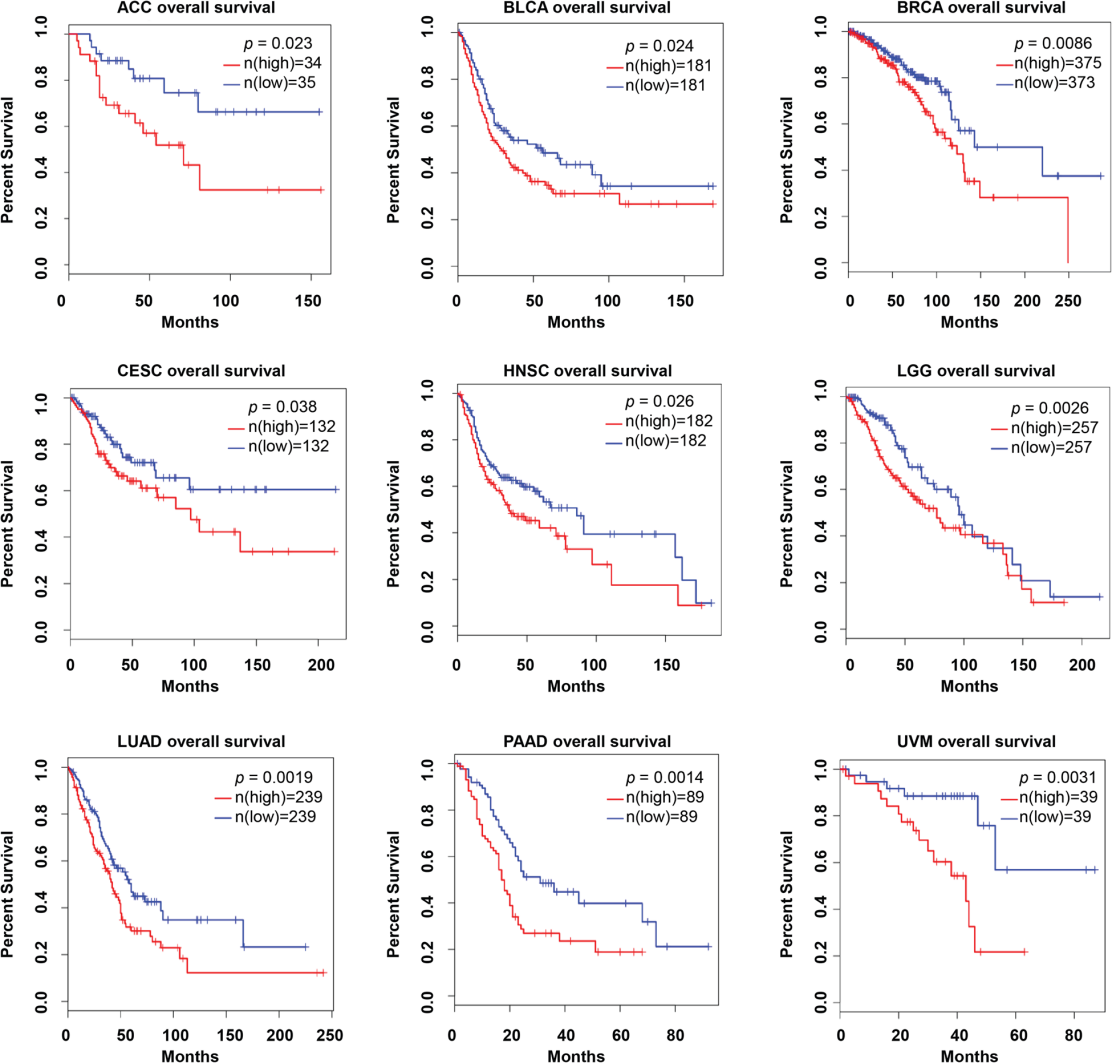
FBXO22 expression is associated with poor survival in a variety of human cancers. Blue line: low FBXO22; red line: High FBXO22. The cutoff-high (%) and cutoff-low (%) for ACC, BLCA, BRCA, CESC, HNSC, LGG, LUAD, PAAD, and UVM are 55/45, 55/45, 65/35, 55/45, 65/35, 50/50, 50/50, 50/50, and 50/50, respectively. ACC adrenocortical carcinoma, BLCA bladder urothelial carcinoma, BRCA breast invasive carcinoma, CESC cervical squamous cell carcinoma and endocervical adenocarcinoma, HNSC head and neck squamous cell carcinoma, LGG brain lower grade glioma, LUAD lung adenocarcinoma, PAAD pancreatic adenocarcinoma, UVM uveal melanoma.

To better clarify the expression pattern and the correlation with patient survival of FBXO22 in human cancers, we examined FBXO22 mRNA expression using the data obtained from the TCGA database. The mRNA levels of FBXO22 were significantly increased in various types of human tumor tissues compared to that in normal tissues. The long list includes bladder urothelial carcinoma (BLCA), breast invasive carcinoma (BRCA), cervical squamous cell carcinoma and endocervical adenocarcinoma (CESC), cholangio carcinoma (CHOL), colon adenocarcinoma (COAD), esophageal carcinoma (ESCA), glioblastoma multiforme (GBM), head and neck squamous cell carcinoma (HNSC), liver hepatocellular carcinoma (LIHC), lung adenocarcinoma (LUAD), lung squamous cell carcinoma (LUSC), rectum adenocarcinoma (READ), thyroid carcinoma (THCA), stomach adenocarcinoma (STAD), and uterine corpus endometrial carcinoma (UCEC) (Fig. 2). Moreover, high levels of FBXO22 were significantly associated with short overall survival. The long list includes adrenocortical carcinoma (ACC), BLCA, BRCA, CESC, HNSC, brain lower grade glioma (LGG), LUAD, pancreatic adenocarcinoma (PAAD), and uveal melanoma (UVM) (Fig. 3). These results imply FBXO22 containing oncogenic characteristics.

## Conclusion and future perspectives

In conclusion, FBXO22 is critically involved in oncogenesis through degradation of multiple substrates (Table 1 and Fig. 1). FBXO22 exerts its tumor promoting role in HCC, lung cancer, breast cancer, but inhibits migration and metastasis in lung cancer and breast cancer, indicating FBXO22 either acting as a tumor suppressor or acting as an oncogene. Thus, there are many fundamental questions that should be addressed to fully understand the role of FBXO22 in tumorigenesis. For example, what are functions of FBXO22 in other types of human cancers other than HCC, lung cancer, breast cancer? What are unknown substrates of FBXO22 that are pivotal in carcinogenesis? What are new regulatory factors to control the expression of FBXO22 in human cancer cells? To answer these questions, it is required to use the FBXO22 knockout or knockin mice to further validate the in vitro data. How can we discover the FBXO22 inhibitors for FBXO22 suppression? A complementary chemical and genomic screening approach might be a novel strategy for achieving FBXO22 inhibitors for treating cancer patients. Without a doubt, more investigations are essential to determine the underlying molecular mechanism of FBXO22-mediated tumorigenesis.
